# ACAT1 Induces the Differentiation of Glioblastoma Cells by Rewiring Choline Metabolism

**DOI:** 10.7150/ijbs.96651

**Published:** 2024-10-14

**Authors:** Shen You, Ming-Jin Wang, Zhen-Yan Hou, Wei-Da Wang, Zhi-Hui Zhang, Ting-Ting Du, Shu-Ying Li, Yi-Chen Liu, Ni-Na Xue, Xiao-Min Hu, Xiao-Guang Chen, Ming Ji

**Affiliations:** 1 Department of Pharmacology, State Key Laboratory of Bioactive Substances and Functions of Natural Medicines, Institute of Materia Medica, Chinese Academy of Medical Sciences and Peking Union Medical College, Beijing 100050, China.; 2 Biomedical Engineering Facility of National Infrastructures for Translational Medicine, Peking Union Medical College Hospital, Chinese Academy of Medical Sciences and Peking Union Medical College, Beijing 100730, China.; 3 Department of Pharmacy, Peking University Third Hospital, Beijing 100080, China.; 4 State Key Laboratory of Complex Severe and Rare Diseases, Peking Union Medical College Hospital, Chinese Academy of Medical Science & Peking Union Medical College, Beijing 100730, China.; 5 Beijing Key Laboratory of New Drug Mechanisms and Pharmacological Evaluation Study, Institute of Materia Medica, Chinese Academy of Medical Sciences and Peking Union Medical College, Beijing 100050, China.; 6 Key Laboratory of Small Molecule Immuno-Oncology Drug Discovery, Chinese Academy of Medical Sciences, Beijing 100050, China.

**Keywords:** glioblastoma, cell differentiation, acetyl coenzyme A acetyltransferase, choline

## Abstract

Abnormal differentiation of cells is a hallmark of malignancy. Induction of cancer-cell differentiation is emerging as a novel therapeutic strategy with low toxicity in hematological malignances, but whether such treatment can be used in solid tumors is not known. Here, we uncovered a novel function of acetyl coenzyme A acetyltransferase (ACAT1) in regulating the differentiation of glioblastoma (GBM) cells. Inhibition of ACAT1 promoted the differentiation of GBM cells into astrocytes but also delayed tumor growth. Mechanistically, suppression of ACAT1 restored mitochondrial function and led to metabolic “reprogramming” in GBM cells: reduction of fatty-acid oxidation and acetyl-CoA, but an increase in free fatty acids. Importantly, ACAT1 negatively regulated the choline metabolic pathway, which is crucial for the differentiation of GBM cells. Finally, we demonstrated that a naturally available substance, chlorogenic acid (CHA), could inhibit phosphorylation of ACAT1 and so delay GBM progression, CHA is a promising candidate to treat GBM because it could induce the differentiation of cancer cells.

## 1. Introduction

Gliomas originate as carcinomas of glial cells. Gliomas are characterized by a high risk of morbidity, recurrence, and mortality 1. Glioblastoma (GBM) is the most aggressive type of glioma. It is characterized by rapid infiltration into nearby brain tissue and resistance to chemotherapy 2. Advanced gliomas have a high mortality rate. The 5-year survival rate for patients with GBM is 4.7% 3.

Standard care includes maximal resection followed by radiotherapy and adjuvant chemotherapy with temozolomide 4. However, long-term survival is rare, with a median overall survival of only 14.6 months with surgery or radiotherapy 5, 6. Therefore, there is an urgent need to study and improve treatment strategies.

“Induced differentiation” therapy differs from radiotherapy in that it is the differentiation of malignant cells to normal cells and mature cells in the presence of “differentiation inducers” 7. There have been many reports of the benefit of differentiation-inducing strategies in the treatment of acute promyelocytic leukemia (APL), particularly all-*trans* retinoic acid (ATRA). The latter is a differentiation agent that has revolutionized the management of a rare APL subtype 8. Over the past 20 years, ATRA has reduced the 5-year mortality rate for APL from 82% to 36% 9. However, this approach has not been achieved in solid tumors.

The abnormal lipid metabolism in most tumor cells is mainly manifested as increased lipid synthesis (including fatty acids) and decreased decomposition of fatty acids. Fatty-acid oxidation (FAO) is an important energy source, producing six-times more adenosine triphosphate (ATP) per unit mass than glycogen decomposition. The activity of FAO has been shown to contribute to aerobic respiration in GBM, whereas expression of the enzymes involved in FAO has been found to be upregulated in human glioma tissues 10. In mitochondrial metabolism, FAO has been found to be related to radiotherapy resistance of nasopharyngeal carcinoma, breast cancer, and glioma 11.

Acetyl coenzyme A acetyltransferase (ACAT1) is expressed specifically in mitochondria, and plays an important part in the production of acetyl coenzyme A (AcCoA) in FAO. As a tetrameric enzyme in ketogenesis, it converts two molecules of AcCoA into acetyl acetyl coenzyme A and coenzyme A, and tetramer formation of ACAT1 from monomers increases its activity 12-15. Studies have shown that phosphorylation of ACAT1 at the residual of Tyr407 could make the ACAT1 tetramer more stable, thereby disrupting the balance between the monomer and tetramer. This action leads to a shift that promotes spontaneous formation of the tetramer, finally increasing ACAT1 activity and promoting the Warburg effect and tumor growth 16. Some reports have shown that phosphorylation of the E1α subunit of pyruvate dehydrogenase (PDH) (encoded by the Pdha1 gene) could limit flux through the PDH complex to reduce glucose oxidation 17. ACAT1 acetylates pyruvate dehydrogenase phosphatase1 (PDP1) at the K202 site and PDHA1 at the K321 site, respectively, leading to the acetylation of PDP1 from PDHA1 dissociation and introduction of active pyruvate dehydrogenase kinase 1 (PDK1) into PDHA1. Also, inhibition of PDC promotes metabolic changes that make cells more dependent upon glycolysis in these cells, whereas knockdown of ACAT1 expression makes cells more dependent on oxidative phosphorylation (OXPHOS) for ATP production 16. Directed OXPHOS plays a central part in the differentiation of normal cells. Fan *et al.* identified a central role for metabolic reprogramming and mitochondrial biogenesis in regulating the differentiation of cancer cells 18, 19.

Choline is an essential nutrient for cell-membrane integrity, transmembrane signaling, phosphatidylcholine (PC) synthesis, neurotransmission, and methyl metabolism 20. Metabolites in the choline metabolic pathway each have their own functions. PC repairs damaged neurons. Cytidine-5'-diphosphate choline (CDP-choline (CDPCho) or CDPC) is the precursor of Phosphatidyl choline (PTD-CHO), which is an important component of neuronal membranes. It is used widely to treat injuries to the central nervous system and diseases 21. In most cell types, PC is synthesized primarily *via* the CDP-choline (Kennedy) pathway in three steps: (1) choline phosphorylation by choline kinase; (2) binding of cytidine triphosphate (CTP) to choline phosphate: choline phosphate cytidylyltransferase (CCT); (3) synthesis of PC from CDP-choline and diacylglycerol (DAG) *via* choline/ethanolamine phosphotransferase 1 (CEPT1) and choline phosphotransferase 1 (CHPT1) 22.

We aimed to explore a novel mechanism of induced differentiation for GBM therapy. Here, we report that ACAT1 drives glioma differentiation by regulating choline metabolism. Our data suggest that the ACAT1 would be a promising target for differentiation-inducing therapy. We also identified a naturally occurring compound, chlorogenic acid (CHA), that can target ACAT1 to interfere with the ACAT1-FAO-PC axis, thereby regulating GBM differentiation.

## 2. Results

### 2.1. Disruption of ACAT1 induced the differentiation of GBM cells

**Figure 1:**
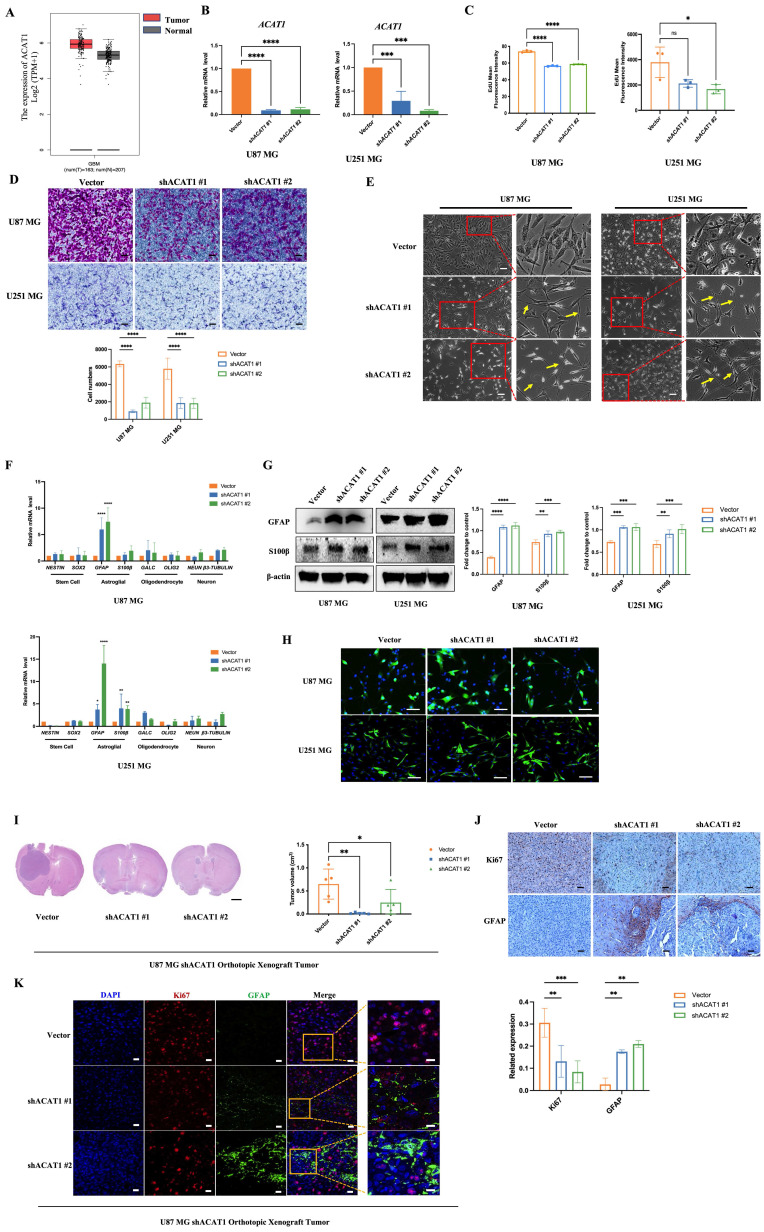
** ACAT1 KD in GBM cells leads to differentiation into astrocytes.** (A) GEPIA data indicated that ACAT1 had high expression in glioma. (B) ACAT1 expression was knocked down at mRNA levels in U87 MG and U251 MG cells. (C) Flow cytometry was used to determine the percentage of EdU cells proliferating after ACAT1 KD in GBM cells. (D) Representative images of invaded cells after ACAT1 KD in GBM cells. Scale bar, 100 μm. (E) Effect of ACAT1 KD on the morphology of U87 MG and U251 MG cells. Microscopy images were captured. Scale bar, 100 μm. (F) Relative mRNA expression of typical markers in stem cells, astrocytes, oligodendrocytes, and neurons of two ACAT1 KD cell lines were detected by real-time RT-qPCR. (G) Western blots of GFAP and S100b in ACAT1 KD GBM cells. β-actin is used as a control. (H) Immunofluorescence analyses of GFAP expression after ACAT1 KD (green). DAPI is shown in blue. Scale bar, 200 μm. (I) The striatum of Balb/c nude mice was inoculated with U87 MG shACAT1 cells (n = 5/group), and two mice were selected randomly from each group to be killed for H&E staining, scale bar, 1000 μm. Tumor volume was observed by NMR imaging, RadiAntViewer software was used to quantify tumor size. (J) Expression of Ki67 and GFAP was analyzed by immunohistochemistry, scale bar, 100 μm. (K) Expression of Ki67 and GFAP was analyzed by mIHC, scale bar, 20 μm. Region of interest, scale bar, 10 μm.

ACAT1 is one of the rate-limiting enzymes in ketogeneses. It has been reported to promote the Warburg effect and tumor growth in lung cancer. However, its role in brain cancers is not known. By using the Gene Expression Profiling Interactive Analysis (GEPIA), an Internet-based tool containing high-throughput RNA-sequencing data (The Cancer Genome Atlas and Genotype-Tissue Expression databases), we found that ACAT1 had high expression in patients suffering from glioma (Figure 1A). This observation triggered exploration of the biological function of ACAT1 in glioma. We employed two human GBM cell lines (U87 MG and U251 MG) and obtained their stable cell lines with knockdown of ACAT1 expression (hereafter termed “ACAT1 KD”) (Figure 1B, Supplementary Figure 1C). As expected, the proliferation and migration capabilities of GBM cells were repressed markedly in ACAT1 KD cells (Figure 1C-D; Supplementary Figure 1A-B). Cell morphology was altered, including stretching, flattening, and growth of protrusions, in U87 MG and U251 MG cells with ACAT1 KD (Figure 1E). Also, serial markers of neurons, astrocytes, oligodendrocytes, and stem cells in the brain were observed by mRNA quantification (Figure 1F). Silencing of ACAT1 expression led to upregulation of expression of the astrocyte markers GFAP and S100β. Immunoblotting also confirmed that the protein expression of GFAP was increased in U87 MG and U251 MG cells with ACAT1 KD (Figure 1G-H). Correspondingly, stable cell lines overexpressing ACAT1 showed decreased GFAP expression and accelerated proliferation, suggesting a critical role for ACAT1 in GBM cells (Supplementary Figure 1D-E).

Next, we confirmed this observation *in vivo*. An orthotopic xenograft of U87 MG cells in the absence of ACAT1 in nude mice was employed to evaluate the differentiation effect of glioma. ACAT1 KD could delay tumor growth in the brain (Figure 1I, Supplementary Figure 1F). Immunohistochemical analyses showed a significant decrease in Ki67 expression and a significant upregulation of GFAP expression in tumors of the shACAT1 group compared with that in the vector group (Figure 1J). Multiplex immunohistochemistry (mIHC) was used to measure the expression of GFAP and Ki67. GFAP had low expression in the vector group, and Ki67 expression was decreased in regions with high expression of GFAP. These data implied that ACAT1 was involved in the differentiation of GBM cells.

### 2.2. ACAT1 KD changed the structure and function of mitochondria

**Figure 2:**
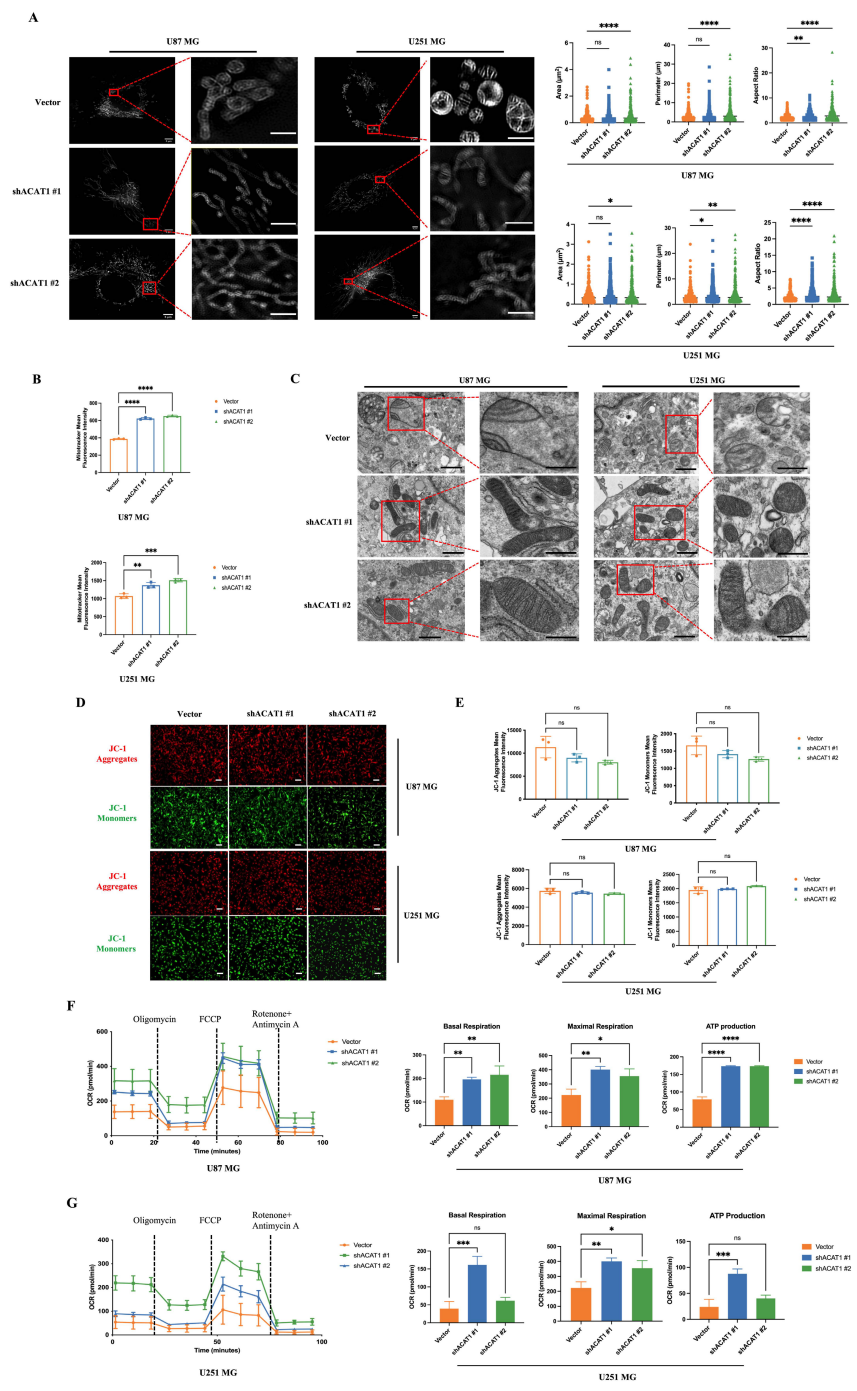
** Effect of ACAT1 KD on mitochondrial structure and function in GBM cells.** (A) Multi-modal structured light super-resolution microscopy (multi-SIM) was used to observe mitochondrial morphology. Scale bar, 5 μm. Region of interest, scale bar, 2 μm. (B) The red fluorescence of MitoTracker in ACAT1 KD GBM cells was analyzed by flow cytometry. (C) Changes in mitochondrial cristae structures in ACAT1 KD GBM cells under transmission electron microscopy. Scale bar, 1 μm. Region of interest, scale bar, 0.5 μm. (D-E) The mitochondrial membrane potential of GBM cells after ACAT1 KD was observed by flow cytometry and fluorescence microscopy. The mitochondrial membrane potential was determined by JC-1 staining. Red represents JC-1 aggregates; Green represents the JC-1 monomers. Scale bar, 100 μm. (F-G) OCR was monitored in real time using an extracellular flux analyzer. Quantitative analysis of basal respiration, maximum respiration, and ATP production was undertaken.

Mitochondria are the “energy factories” of cellular life activities. They are the sites of OXPHOS for ATP synthesis. ACAT1 is expressed specifically in mitochondria. Next, we investigated if mitochondrial structure and function was changed after ACAT1 abolishment. The morphological changes of mitochondria were observed by multi-modal structured light super-resolution microscopy (multi-SIM) (Figure 2A). The area and perimeter of mitochondria in ACAT KD cells became larger, and the aspect ratio increased significantly, changing from spherical to linear. Mitochondrial numbers were detected *via* a fluorescent dye (MitoTracker™ Red). The mitochondrial number was altered significantly in cells with ACAT1 KD (Figure 2B). These results indicated that GBM cells tended to be more active in energy metabolism after ACAT1 KD. Transmission electron microscopy showed that the arrangement of mitochondrial cristae structures in ACAT1 KD cells was more uniform and the number also increased to a certain extent (Figure 2C). Then, we examined the changes in mitochondrial membrane potential using a fluorescent lipophilic carbocyanine dye: JC-1. We documented a certain degree of decrease in cell membrane potential after ACAT1 KD, but the difference was not significant (Figure 2D-E).

Furthermore, we measured the oxygen consumption rate (OCR) to verify whether mitochondrial function was restored in GBM cells in the absence of ACAT1. We noticed a significant increase in the OCR in ACAT1 KD cells, including an increase in basal oxygen consumption rate and ATP production (Figure 2F-G). These data implied that abolishment of ACAT1 could restore mitochondrial structure and function by regulating metabolic reprogramming, thus contributing to the differentiation of GBM cells.

### 2.3. ACAT1 repressed the choline metabolism pathway

**Figure 3:**
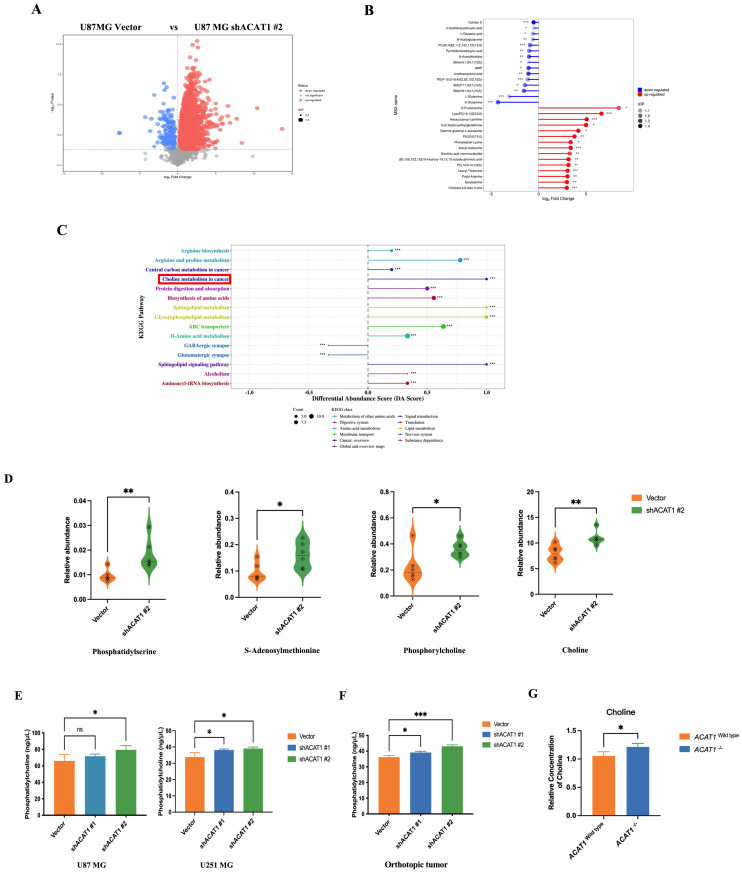
** ACAT1 KD activates choline metabolism in GBM cells.** (A) Volcano maps comparing differential metabolites in ACAT1 KD GBM cells. (B) Matchstick analysis for the metabolites of ACAT1 KD in GBM cells with upregulated and downregulated expression. (C) Pathway analyses of ACAT1 KD differentially enriched metabolites in GBM cells. (D) Differential metabolites in the choline metabolic pathway. (E) PC levels were examined in U87 MG and U251 MG cells of shACAT1. (F) PC levels were examined in U87 MG orthotopic tumor tissues. (G) Changes in choline levels in the brain of *ACAT1^-/-^* and* ACAT1 ^wild type^ mice*.

The roles of ACAT1 in the metabolism of fatty acids and ketones have been reported widely. We speculated that the differentiation of GBM cells after ACAT1 KD may be related to metabolic reprogramming. To ascertain the underlying mechanism of induced differentiation of GBM cells, we undertook metabolomics analysis in U87 MG cells. Differentially expressed metabolites were first screened using principal component analysis (PCA) and partial least squares discriminant analysis (PLS-DA). Compared with the vector group, ACAT1 KD in positive-ion mode elicited 224 metabolites with differential expression. PCA revealed significant differences between the two groups of principal components (Figure 3A, Supplementary Figure 2A-B). Notably, expression of metabolites that promote tumor-cell proliferation (e.g., glutamine) was downregulated (Figure 3B).

We subjected these differential metabolites to analysis of signaling-pathway enrichment using the KEGG database. The “Sphingolipid signaling pathway”, “Glycerophospholipid metabolism”, and “choline metabolism pathway” were activated significantly. The “GABAergic synapse” and “Glutamatergic synapse pathways”, which promote glioma proliferation, were inhibited significantly (Figure 3C), which might be related to the role of ACAT1 in fatty-acid metabolism. Then, we focused on activation of the choline metabolic pathway (choline is a component of biological membranes and a prerequisite for acetylcholine synthesis). Expression of choline, PC, phosphatidylserine, phosphatidylethanolamine, and S-adenosylmethionine was upregulated (Figure 3D). Citicoline and PC in this pathway repair damaged nerves, so we hypothesized that choline metabolism may be more relevant to the differentiation of GBM cells.

To test this hypothesis, we measured the PC level *in vitro* and *in vivo*. The PC level increased upon ACAT1 KD in U87 MG and U251 MG cells (Figure 3E). The PC level in the tumor issues of ACAT1 KD animal group was also increased significantly (Figure 3F). In addition, *ACAT1^-/-^* mice from our research team showed a higher concentration of choline in hippocampal and cortical regions according to nuclear magnetic resonance (NMR) imaging compared with that in *ACAT1^wild type^* mice (Figure 3G, Supplementary Figure 2C-D).

### 2.4. Activation of the choline metabolic pathway contributed to the differentiation of GBM cells

**Figure 4:**
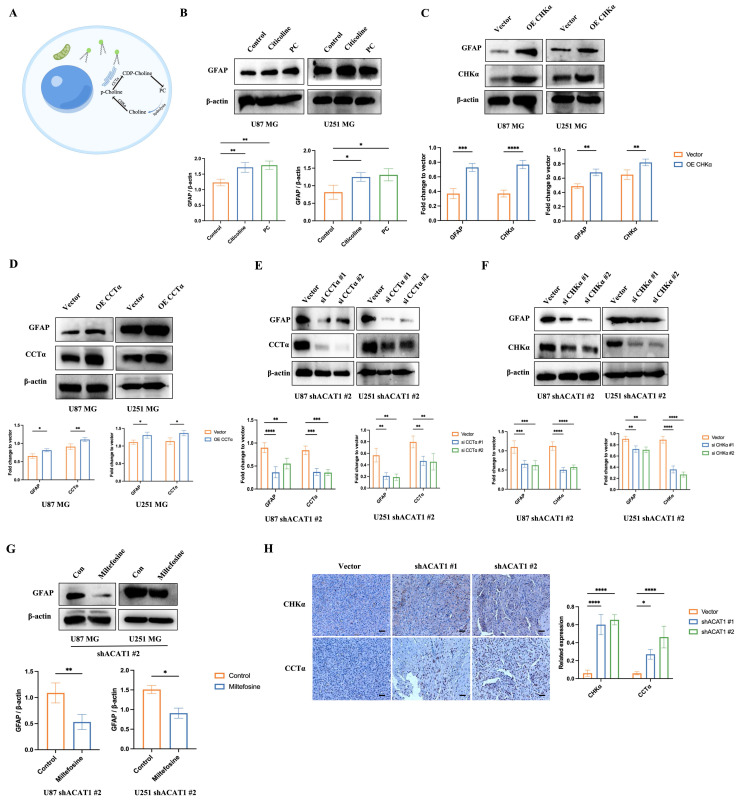
** Activation of the choline metabolic pathway promotes the differentiation of GBM cells.** (A) Choline metabolic pathway (schematic). (B) GBM cells were treated with citicoline (10 μM) and PC (0.1 μM), and GFAP was detected by western blotting. (C-D) Overexpression of CHKα and CCTα in GBM cells, and western-blot analysis of changes in GFAP, CHKα, and CCTα in GBM cells. (E-F) Silent expression of CHKα and CCTα in the construct of shACAT1 #2 stably transfected cell lines, and western-blot analysis of changes in GFAP, CHKα, and CCTα. (G) Treatment of GBM cells with an inhibitor of CCTα, miltefosine (10 μM), shACAT1 #2 stably transfected cell line, and western-blot analysis of GFAP changes. (H) Expression of CHKα and CCTα in U87 MG orthotopic tumor tissues was analyzed by immunohistochemistry. Scale bar, 100 μm.

Three metabolic pathways in cells have been reported in choline production. Among these, activation of choline kinase α (CHKα) and CCT can activate the choline pathway (Figure 4A). We assumed that the terminal product of this pathway, PC, might contribute to the differentiation of GBM cells. Hence, we tested whether add PC in GBM cells or activation of the choline pathway would promote the differentiation of GBM cells. First, we supplemented PC and citicoline (precursor of PC) in U87 MG and U251 MG cells: PC and citicoline resulted in upregulation of the astrocyte marker GFAP (Figure 4B).

Furthermore, overexpression of CHKα and CCT in GBM cells upregulated the expression of GFAP, which suggested that activation of the choline metabolic pathway drives the differentiation of GBM cells (Figure 4C-D). Conversely, GFAP expression was downregulated significantly after knockdown of CHKα and CCT by small interfering (si)RNA in U87 MG and U251 MG cells in the absence of ACAT1 (Figure 4E-F). Similar results were observed in the presence of an inhibitor of CCT synthesis: miltefosine (Figure 4G). Consistent with the results at the cellular level, the results of immunohistochemistry showed that expression of CHKα and CCT was upregulated in the ACAT1 KD animal group (Figure 4H). These results suggested that CHKα and CCT were involved in the cholinergic pathway that regulates the differentiation of GBM cells.

### 2.5. ACAT1 negatively regulated PC production in GBM cells by affecting fatty-acid metabolism

**Figure 5:**
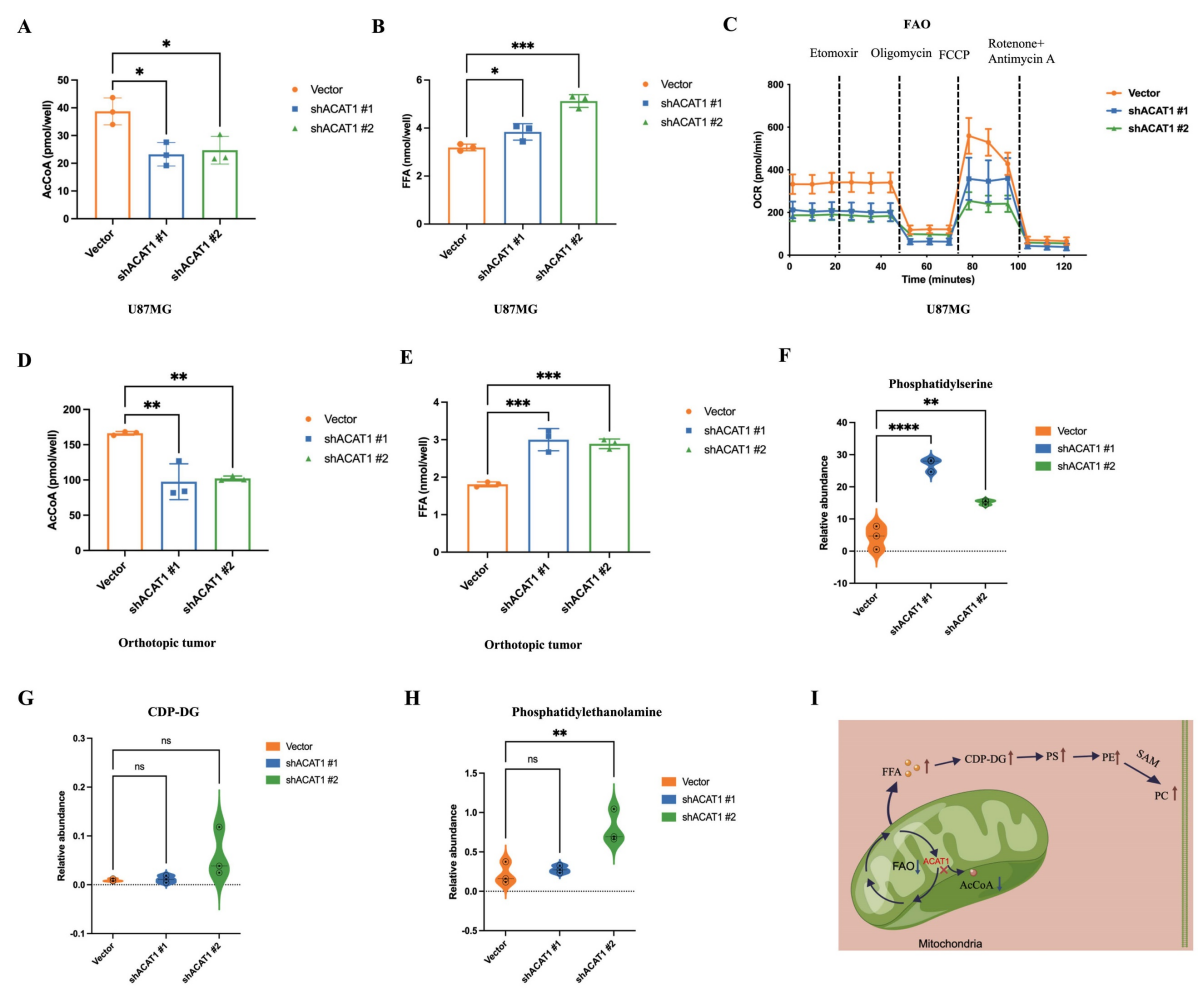
** ACAT1 restrained the generation of PC by adjusting FAO.** (A-B) AcCoA and FFAs were detected in U87 MG cells of shACAT1. (C) FAO was examined by an extracellular flux analyzer. (D-E) AcCoA and FFAs were detected in orthotopic tumors. (F-H) PS, CDP-DG, and PE were measured by MS. (I) ACAT1 knockdown promoted the PC pathway (schematic).

We demonstrated that activation of the choline metabolic pathway could promote the differentiation of GBM cells. Hence, we further explored how the choline metabolism pathway was activated in GBM cells after ACAT1 KD. ACAT1 has the role of transacetylation in the final step of FAO to generate AcCoA. Thus, we measured the change in the concentrations of AcCoA in U87 MG cells after disruption of ACAT1. As expected, the concentration of AcCoA was significantly lower in the ACAT1 KD group compared with that in the vector group (Figure 5A). Meanwhile, the concentration of free fatty acids (FFAs) was significantly higher in the ACAT1 KD group (Figure 5B). We used OCR to assess the changes in the FAO rate in U87 MG cells. The data suggested that the rate of FAO decreased significantly after ACAT1 KD compared with that in the vector group (Figure 5C). Consistently, FFA accumulated in tumor tissues in the ACAT1 KD group, and the level of AcCoA was lower in the ACAT1 KD group than that in the vector group (Figure 5D-E). Taken together, these results indicated that ACAT1 boosted cellular FAO and blocked FFA accumulation.

Due to the higher concentration of FFAs, we hypothesized that the increased synthesis of PC might be due to activation of the pathway of CDP-diacylglycerol to phosphatidylethanolamine. Then, we verified this concept through mass spectrometry (MS). MS showed that as the production of CDP-DG, phosphatidylserine, and phosphatidylethanolamine in the shACAT1 #1 and #2 of U87 MG cell group increased significantly (Figure 5F-H). This observation was basically consistent with the differential metabolites identified by metabolomics analysis. The results stated above indicated that the ACAT1-FAO-PC axis had an important role in the differentiation of GBM cells (Figure 5I).

### 2.6. The naturally occurring molecule CHA promoted the differentiation of GBM cells by inhibiting the enzyme activity of ACAT1

**Figure 6:**
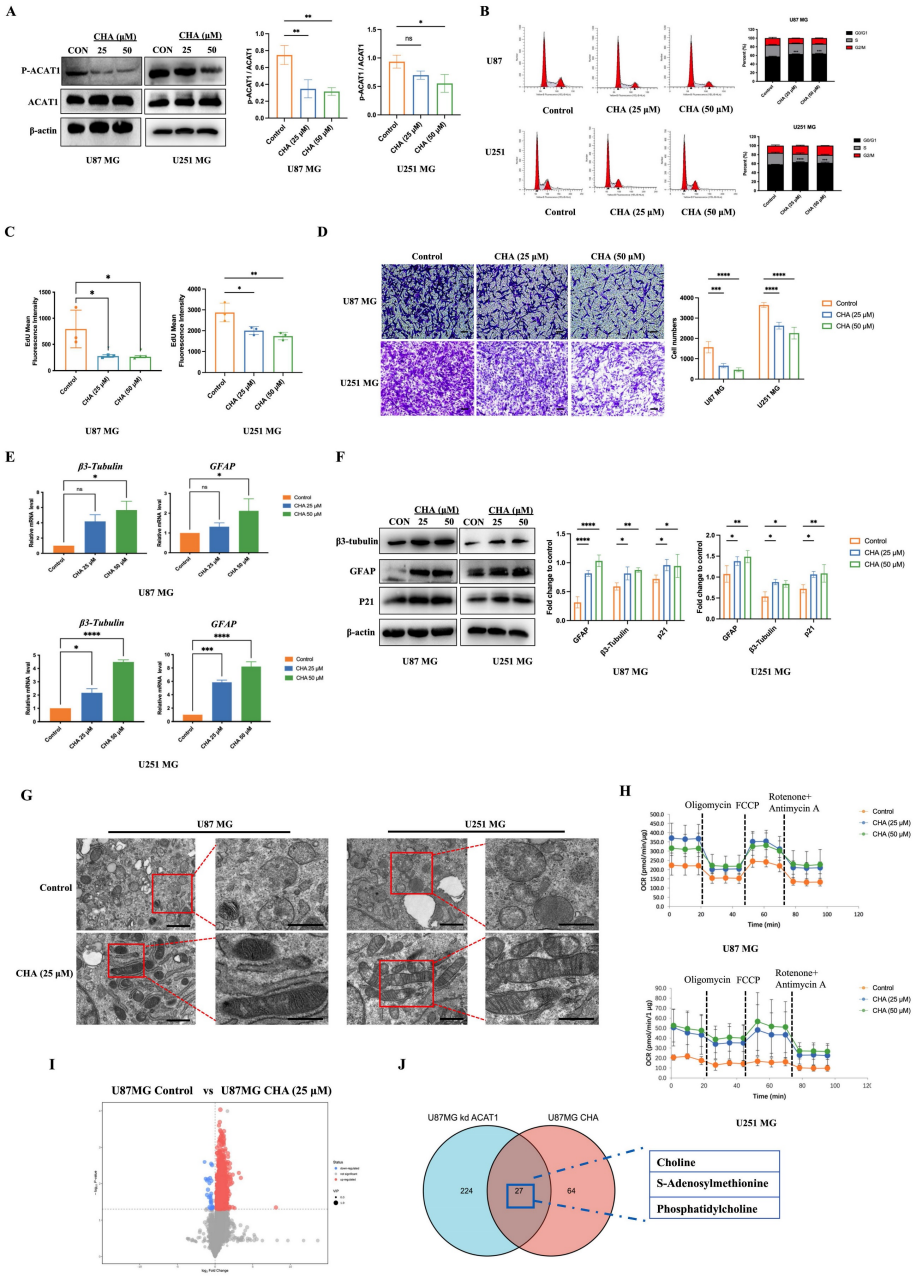
** CHA regulated the differentiation of GBM cells by inhibiting p-ACAT1 (Tyr407).** (A) GBM cells were treated with CHA (25 μM or 50 μM). After 24 h, western blotting was done to ascertain the change in p-ACAT1 (Tyr407). (B) Cell-cycle arrest occurred in G0/G1 phase in GBM cells treated with CHA (25 μM or 50 μM) for 24 h. (C) The percentage of proliferating EdU-positive cells were measured by flow cytometry, and both cell types were treated with CHA (25 μM or 50 μM) for 7 days. (D) Representative images of invasive cells treated with CHA (25 or 50 μM) for 7 days. Scale bar, 100 μm. (E) Relative mRNA expression of GFAP and β3-tubulin in two cell lines treated with CHA (25 μM or 50 μM) for 24 h were detected by real-time RT-qPCR. (F) Western blots of GFAP, β3-tubulin, and p21 in GBM cells treated with CHA (25 μM or 50 μM) for 24 h. (G) Transmission electron microscopy of GBM cells treated or mot treated with CHA. Scale bar, 1 μm. Region of interest, scale bar, 0.5 μm. (H) OCR assay was determined using GBM cells treated with CHA (25 μM or 50 μM) for 7 days. (I) Volcano maps were used to analyze the differential metabolites of GBM cells treated with CHA (50 μM) for 7 days. (J) Venn diagram representing 27 identical differential metabolites between GBM cells treated with CHA and ACAT1 KD.

Previous studies from our research team have shown that CHA can interact with ACAT1 and inhibit the enzyme activity of ACAT1. It has been reported that the phosphorylation of ACAT1 at the residue Tyr407 enhances ACAT1 activity 16, 23, 24. We found that CHA could reduce the phosphorylation level of ACAT1 in GBM cells (Figure 6A). Long-term treatment with CHA could inhibit the proliferation of GBM cells and block the cycle of GBM cells at the G0/G1 phase (Figure 6B). 5-ethynyl-2'-deoxyuridine (EdU) staining showed that the intensity of fluorescence was decreased significantly after CHA treatment, indicating that CHA inhibited the proportion of proliferating GBM cells (Figure 6C). We also examined the changes in expression of markers associated with the differentiation of GBM cells at transcriptional and protein levels after CHA treatment. Real-time reverse transcription-quantitative polymerase chain reaction (RT-qPCR) and western blotting consistently showed upregulation of expression of GFAP and β3-tubulin, as well as diminished cell migration after differentiation (Figure 6D-F).

CHA had no effect on the cell membrane potential (Supplementary Figure 3A), indicating that the inhibition of cell proliferation by CHA was through the induction of a differentiation pathway rather than cytotoxicity. Transmission electron microscopy confirmed the normalization of the cristae structure of mitochondria after CHA-induced differentiation (Figure 6G). This observation was consistent with the results in GBM cells with ACAT1 KD, where we also confirmed the recovery of OXPHOS after long-term induction with CHA (Figure 6H, Supplementary Figure 3B-C). We also undertook metabolomic analysis of U87 MG cells after CHA-induced differentiation. There were 64 differential metabolites in U87 MG cells after CHA induction compared with those in the control group (Figure 6I-J, Supplementary Figure 3G). Of these, 27 differential metabolites were consistent with those in U87 MG cells after ACAT1 KD. Analyses of signaling-pathway enrichment using the KEGG database revealed the “Activated choline metabolic pathway” was enriched (Supplementary Figure 3H). These results suggested that activation of the choline metabolic pathway could induce the differentiation of GBM cells toward astrocytes.

### 2.7. CHA induced inhibition of the proliferation and differentiation of tumor cells in a xenograft model

**Figure 7:**
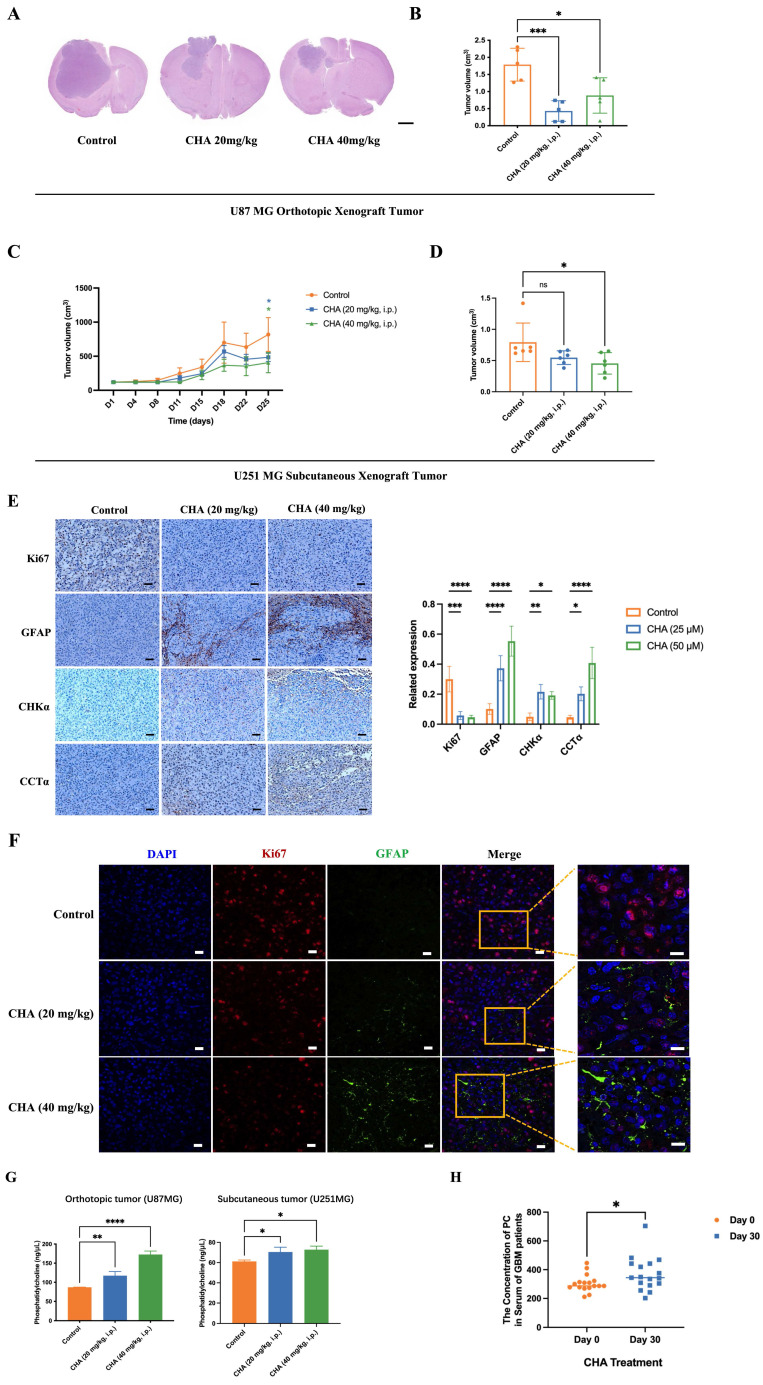
** CHA promoted the differentiation of GBM cells *in vivo* and activated choline metabolism.** (A-B) Balb/c nude mice were inoculated with U87 MG cells in the striatum (n =5/group). Three days after inoculation, mice were injected intraperitoneally with vehicle or CHA (20/40 mg/kg/d). We measured tumor size through MRI and killed mice at the endpoint (bodyweight of mice was decreased by 20%) for H&E staining of a brain cross-sections, Scale bar, 1000 μm. (C-D) Balb/c nude mice were inoculated subcutaneously with U251 MG cells (n = 6/group). Mice were injected intraperitoneally with CHA (20/40 mg/kg/d). Bodyweight and tumor volume (mean ± SD) were recorded every 3 days for subcutaneous inoculation. (E) Immunohistochemical analyses for the expression of Ki67, GFAP, CHKα and CCTα. Scale bar, 100 μm. (F) Expression of Ki67 and GFAP was analyzed by mIHC, scale bar, 20 μm. Region of interest, scale bar, 10 μm. (G) PC levels were determined in tumor tissues treated with CHA (20/40 mg/kg, i.p.). (H) Patients with glioma were treated with CHA for 30 days and their serum were used to determine changes in PC levels. Data are the mean ± SD (n = 17 per group).

We showed that CHA could induce the differentiation of GBM cells. Next, we evaluated the effect of CHA *in vivo*. U87 MG cells were used to establish an orthotopic xenograft model in nude mice. U251 MG cells were used to establish a subcutaneous xenograft model in nude mice. The effects of CHA on the proliferation and differentiation of tumor cells were evaluated, respectively. The orthotopic inoculation model and subcutaneous inoculation model demonstrated that CHA could inhibit the growth of glioma and increase the bodyweight of mice significantly (Figure 7A-D, Supplementary Figure 4A-D), indicating that CHA acted as a differentiation inducer and was not toxic. Treatment with CHA could reduce the expression of Ki67 in the tumor and increase the expression of GFAP, with increase the expression of CHKα and CCTα (Figure 7E). Next, we evaluated whether there was a change in the PC concentration in the tumor after CHA treatment. Compared with the control group, the CHA treatment group had significantly increased PC content (Figure 7F). Furthermore, the PC content in the serum of patients with grade-IV GBM, who are currently receiving a CHA injection in a clinical trial (NCT03758014), was measured. The PC concentration in patient serum was increased significantly after CHA treatment (Figure 7G). These results indicated that CHA could activate the choline metabolic pathway, leading to the differentiation of GBM cells.

## 3. Discussion

ACAT1, as a metabolic enzyme, is involved in tumor progression. However, the relationship between ACAT1 and the differentiation of GBM cells has not been investigated. Herein, we clarified the mechanism of differentiation from the perspective of tumor metabolism. Silencing of ACAT1 expression enabled the accumulation of FFAs by decreasing the rate of FAO which, in turn, activated the choline metabolic pathway. An increased level of the metabolic end-product PC contributed to the differentiation of glioma cells towards astrocytes. This is the first report of the relationship between choline metabolism and glioma differentiation.

ACAT corresponds to two ubiquitous metabolic enzymes localized in the mitochondria and cytoplasm. ACAT1 is expressed specifically in the mitochondria. ACAT2 is expressed specifically in the cytoplasm 25, 26. In addition to the proven ways of isoleucine metabolism, ketogenic metabolism, and FAO, ACAT1 catalysis is also related to cancer. The mutation of ACAT1 can deacetylate PDH and inhibit tumor proliferation, so ACAT1 is regarded as a potential target for anticancer drugs 16, 27. Mitochondria are the center of cellular energy metabolism, but also regulate the epigenetic inheritance of cells by providing AcCoA 28. One of the signs of a tumor is a disorder in energy metabolism. The Warburg effect indicates that tumor cells prefer aerobic glycolysis 29. We investigated the relationship between ACAT1 and mitochondrial function in GBM cells. We also demonstrated that silencing of ACAT1 expression did not impair mitochondrial function, but increased the number of mitochondria, thereby shifting the energy supply of GBM cells from aerobic glycolysis to OXPHOS. Studies have shown that tumor cells with high levels of OXPHOS have a more regular mitochondrial cristae structure, and tumor cells can inhibit growth and promote differentiation after switching from glycolysis to OXPHOS 30-32. We also showed that silencing of ACAT1 expression in GBM cells led to inhibition of tumor proliferation *in vivo* and *in vitro*.

Increased *de novo* synthesis of lipids in tumor cells promotes the need for tumor growth. Expression of key enzymes for lipid synthesis is upregulated in many cancer types 33-35. ACAT1 is a key enzyme in fatty-acid metabolism. It is involved in the catabolism and synthesis of acetylacetyl coenzyme A 36. We revealed the relationship between ACAT1 and AcCoA. That is, the silencing of ACAT1 expression could reduce AcCoA production. It has been shown that the accumulation of lipid droplets occurs in glioblastoma, which increases FAO and promotes tumor proliferation 37. We hypothesized that a decrease in the AcCoA level would lead to a decrease in lipid production, which could slow tumor growth due to an insufficient energy supply. We also used OCR to evaluate long-chain fatty-acid stress. We showed that the FAO rate was reduced significantly after knockdown of ACAT1 expression, suggesting impaired metabolism of fatty acids. An increasing number of studies have shown that lipid metabolism in cancer cells is altered significantly compared with that in normal cells. This is especially true for the increased synthesis of fatty acids, which are the main components of the cell membrane. However, excessive fatty acids can cause severe cytotoxicity, so controlling the level of FFAs is particularly important to maintain cellular homeostasis 38-40. In our study, FFA content increased after silencing of ACAT1 expression in GBM cells. We speculated that lipids could not enter mitochondria for oxidation and decomposition due to a decrease in the FAO rate, which eventually led to lipid accumulation. FFAs include cholesterol, neutral fats, and phospholipids. Therefore, the increase in FFA content activated the glycerophospholipid metabolic pathway. Metabolomics analysis and MS also indicated an increase in levels of CDP-DG, PS, and PE. We hypothesized that the reaction between an increased level of CDP-DG and serine led to increased PS content, and decarboxylation of PS led to PE, which eventually led to PC in the presence of SAM, thereby activating the choline metabolic pathway.

PC is known as the “third nutrient”, along with protein and vitamins. It is an important component of nerve tissue. Pc is an advanced neurotrophin concentrated in the human brain, nervous system, blood circulation system, immune system, heart, liver, lungs, kidneys, and other important organs. It can repair damaged brain cells and improve memory capacity to prevent Alzheimer's disease. Studies have shown that a PC-rich medium enhanced neuronal differentiation, and that PC supplementation promoted neurogenesis by increasing the number of healthy neurons. Furthermore, PC ameliorated neuronal damage and thus modulated neuronal plasticity 41. However, studies on the role of PC in promoting the differentiation of GBM cells are lacking. Our experiments on GBM cells supplemented with PC showed that PC could repair damaged neurons, but also tended to differentiate undifferentiated GBM cells towards mature cells and reduce tumorigenesis and tumor development. The PC-mediated hydrolysis pathway mainly generates choline and phosphatidic acid. Choline is catalyzed by CHKα to generate phosphorylcholine, which enters the Kennedy pathway. The second biosynthetic step of the choline branch of the Kennedy pathway is catalyzed by a specific CCT, which uses phosphorylcholine and CTP to form the high-energy donor CDP-choline and release pyrophosphate, as well as CDP-choline and glycerol diesters to generate PC 42-44. We also verified that activation of the choline metabolic pathway drove GBM cells toward terminal differentiation, and our results strongly supported this view. Some studies have reported that PE can regulate the development of Tfh cells and the humoral immune response by regulating the expression and localization of CXCR5 45. Some studies have shown increased synthesis of PS to be a key metabolic event mediating the oncogenic function of OTUB2. Preclinical results have suggested that PS given *via* the oral route might aid inhibiting the development of tongue and esophageal squamous cell carcinoma 46. PE and PS are products in the choline metabolic pathway, which have potential value in inhibiting tumor proliferation and boosting immunity. Abnormal PC metabolism has been reported to be a hallmark of cancer cells, especially in breast, ovarian, and endometrial cancers 47-49. It has also been shown that promoting LPCAT3-mediated binding of unsaturated lipids to PC can improve the endoplasmic reticulum stress response and impede the pro-tumor phenotype and survival of tumor-associated macrophages 50. We suggest that the lipid accumulation induced by the silencing ACAT1 expression might not be quantitatively sufficient to cause proliferation-promoting tumors. Instead, we hypothesize that the reduced FAO rate resulting in the inability of tumors to rely on lipid metabolism for energy supply has a greater effect on tumor proliferation. PC may have inconsistent effects in different organs, whereas a lower degree of upregulation in GBM cells results in the promotion of GBM-cell differentiation to astrocytes. Our results also supported the speculation made above, and related data suggest that the upregulation of PC content was not robust.

Studies have shown that ATRA can induce leukemia cells to differentiate into normal cells and achieve long-term survival if administered to patients suffering from APL 51. However, no differentiation-inducing agent has been able to achieve satisfactory results in the treatment of solid tumors. Our previous study revealed that the target of CHA was phosphorylated (p)-ACAT1 (Tyr407) 24. Our results also showed that CHA could promote the differentiation of GBM cells by inhibiting p-ACAT1 (Tyr407). Also, a single treatment with CHA did not decrease the mitochondrial membrane potential, indicating that CHA is a safe and efficacious differentiation-inducing agent. CHA has been reported to promote the differentiation of hepatocellular carcinoma and lung cancer by regulating microRNAs 52, but we elucidated a new mechanism from the perspective of CHA regulating ACAT1. Moreover, data from clinical trials have shown that CHA is safe and efficacious, significantly lengthening survival in patients with recurrent high-grade glioma. Also, the increased PC concentration in the serum of patients suggested that CHA could activate choline metabolic pathway and promote the differentiation of GBM cells. These results provided a theoretical basis for further clinical application of this drug.

## 4. Conclusions

We demonstrated the effect of ACAT1 on the differentiation in GBM cells. We elucidated the role of the ACAT1-FAO-PC pathway in controlling the differentiation and growth of tumor cells. However, the molecular mechanism of ACAT1 regulation of FAO is not known. Induced differentiation is an efficacious and safe treatment strategy compared with conventional cancer therapies. Our data support ACAT1 as a target for induced differentiation and a promising strategy for cancer treatment. As a natural small-molecule product, CHA could promote the differentiation of GBM cells by regulating p-ACAT1 Tyr407. CHA could be a new candidate drug for the clinical treatment of glioma.

## 5. Materials and methods

### 5.1. Reagents

Antibodies specific for ACAT1 phosphorylated on Tyr407 were generated by Signalway Antibody (Greenbelt, MD, USA; 1:200 dilution). Other primary antibodies used for western blotting were: anti-GFAP (1:1000; catalog number: 12389; Cell Signaling Technology, Danvers, MA, USA), anti-ACAT1 (1:1000; 44276; Cell Signaling Technology), anti-β3-tubulin (1:1000; 5568; Cell Signaling Technology), anti-P21 (1:1000; 2947; Cell Signaling Technology), anti-Ki67 (1:500; 9449; Cell Signaling Technology), anti-CCTα (1:1000; ab109263; Abcam, Cambridge, UK), anti-choline kinase alpha (1:1000; ab88053; Abcam), anti-S100b (1:1000; 15146-1-AP; Proteintech, Rosemont, IL, USA), anti-β-actin (TA346894; ZSGB-BIO, Beijing, China). CHA was provided by Jiujiang Biochemical Engineering Technology Development (Chengdu, China). For *in vitro* experiments, CHA was dissolved in dimethyl sulfoxide at an appropriate concentration. For *in vivo* experiments, CHA was dissolved in physiologic (0.9%) saline at a desired concentration.

### 5.2. Cell culture

The U87 MG, U251 MG, and HEK293T cell lines were ordered from American Type Cell Culture (Manassas, VA, USA). All cell lines were certified by the respective institutes from which we purchased them. Human glioma U87 MG and U251 MG cells were grown in Dulbecco's modified Eagle's medium. HEK293T cells were grown in minimum essential medium (MEM). Both types of media were supplemented with 10% fetal bovine serum (FBS; heat-inactivated at 56°C for 30 min) and appropriate amounts of penicillin (50 U/mL)/streptomycin (50 mg/mL) in an incubator at 37°C in a humidified environment with 5% CO_2_.

### 5.3. Western blotting and immunohistochemistry

Cells were lysed in lysis buffer containing a complete protease inhibitor cocktail. Protein concentrations were determined using the BCA Protein Assay Kit (Beyotime Biotechnology, Shanghai, China). Equal amounts of proteins were separated by sodium dodecyl sulfate-polyacrylamide gel electrophoresis and transferred to polyvinylidene difluoride (PVDF) membranes. PVDF membranes were blocked in 5% skimmed milk solution in Tris-buffered saline containing 0.1% Tween (TBST) for 1 h and incubated with primary antibody at 4°C overnight. Secondary antibodies were incubated with cells for 1 h. Then, the blots were visualized using a chemiluminescence detection kit (Bio-Rad Laboratories, Hercules, CA, USA).

Immunohistochemistry was undertaken on 2 mm-thick, formalin-fixed, paraffin-embedded slices. After dewaxing, permeabilizing, and blocking, primary and secondary antibodies were incubated sequentially for detection.

### 5.4. mIHC and imaging

Briefly, mIHC staining of brain sections on coverslips was done using anti-Ki67 and anti-GFAP and a multiplex immunohistochemistry/staining kit (NEL810001KT; Opal 3-Plex Manual Detection Kit; Akoya Biosciences, Marlborough, MA, USA) according to manufacturer instructions.

### 5.5. Lentiviral transduction

For the study of overexpression or knockdown of viruses, 5 µL of lentivirus was added to cells for 24 h. Then, the virus was removed and cultured in a medium containing puromycin. The short hairpin (sh)RNA sequence against ACAT1 was purchased from Guangzhou IGE Biotechnology (Guangzhou, China). The target sequence of ACAT1 #1-specific shRNA is 5′- CGAAATGAACAGGACGCTTATCTCGAGATAAGCGTCCTGTTCATTTCG-3′ and the target sequence of ACAT1 #2-specific shRNA is 5′-GCCTTTAGTCTGGTTGTACTACTCGAGTAGTACAACCAGACTAAAGGC-3′. Control (PKO.1-puro) shRNAs were synthesized by Guangzhou IGE Biotechnology. For ACAT1 overexpression, a lentivirus construct expressing the FLAG-tagged coding sequence of human ACAT1 (pLV[Exp]-Puro-CMV>hACAT1[NM_001386677.1]) was generated. pLV[Exp]-Puro virus was used as the control. Transfection was carried out using a transfection reagent (Lipofectamine™ 3000) according to manufacturer (Invitrogen, Carlsbad, CA, USA) instructions.

### 5.6. RNA extraction and real-time RT-qPCR

Total RNA from cultured cells was extracted using Quick RNA Extraction Kit (ES Science, Shanghai, China) according to manufacturer instructions. RNA from each sample (2 μg) was used for complementary (c)DNA synthesis. We used All-in-One First-Strand cDNA Synthesis SuperMix for qPCR (TransGen Biotech, Beijing, China) for reversing cDNA. For real-time RT-qPCR, we used SYBR Green qPCR primer pairs (TransGen Biotech). To detect mRNA, we used the primers shown in Table 1. To ensure reproducibility, all genes were tested in triplicate. Real-time RT-qPCR was done on the ABI PRISM 7900HT sequence-detection system (Thermo Fisher Scientific) using the following cycling parameters: an initial denaturation step of 95°C for 10 min, followed by 95°C for 15 s, 60°C for 10 s, and 72°C for 25 s of 40 cycles.

### 5.7. Migration assay

CHA (25 μΜ or 50 μΜ)-treated cells and ACAT1 KD cells were trypsinized and resuspended in serum-free medium. Next, 100 μL of cell suspension (2 × 10^4^ cells) was seeded into the upper chamber of a Transwell™ apparatus (Corning, Corning, NY, USA), which is a porous polycarbonate membrane (pore size = 8 μm). The lower chamber contained 700 μL of culture medium supplemented with 20% FBS. After 24 h of incubation, cells on the upper surface of the membrane filter were removed with a cotton swab. Migrating cells on the lower side of the filter were fixed with 4% paraformaldehyde, stained with 0.1% crystal violet solution for 15 min, washed with phosphate-buffered saline, air-dried, and imaged under a microscope.

### 5.8. Flow cytometry

Flow cytometry was done on a flow cytometer (Guava easyCyte; Luminex, Austin, TX, USA). For assessment of cell proliferation, cells were incubated with EdU (10 µM; Beyotime Biotechnology) for 2.5 h. EdU was chemically combined with a click reaction solution (Azide 594) for 30 min. JC-1 (Beyotime Biotechnology) was used to assess the change in the mitochondrial membrane potential. Cells were incubated in JC-1 staining working solution for 20 min in a 37°C cell incubator. MitoTracker Red (Beyotime Biotechnology) was used to assess mitochondrial number. Cells were incubated with MitoTracker Green (50 nM) at 37°C for 30 min prior to analyses. For cell-cycle assessment, cells were stained with phosphatidyl ethanolamine (PE) and analyzed by flow cytometry.

### 5.9. CellTiter-Glo (CTG) proliferation assay

CTG proliferation experiments were undertaken according to manufacturer protocols. Briefly, cells were inoculated on 24-well plates with 10,000 cells per well and cultured for 3 days. CTG reagent (Promega, Madison, WI, USA) was added to lysed cells and chemiluminescence was measured with a plate reader (Synergy H1; BioTek, Winooski, VT, USA).

### 5.10. Measurement of cellular metabolism

Cellular OXPHOS and long-chain fatty-acid oxidative stress were monitored by real-time measurement of OCR using the Extracellular Flux Analyzer (XF24; Seahorse Bioscience, North Billerica, MA, USA). Briefly, 10,000 cells were seeded in specific 24-well plates designed for the Extracellular Flux Analyzer. Then, 200 µL of appropriate growth medium was added, followed by overnight incubation. Prior to measurement, cells were washed with XF Base Medium with glucose (10 mM), sodium pyruvate (1 mM), and glutamine (2 mM). Then, 500 mL per well of the medium prepared above was added, followed by incubation for 1 h in the absence of CO_2_. OCR was measured in typical 8-min cycles of mixing (2-4 min), dwelling (2 min), and measurement (2-4 min), as recommended by Seahorse Bioscience. Basal levels of OCR were first recorded, and then levels of OCR after successive addition of relevant inhibitor compounds were recorded.

### 5.11. Quantification of mitochondria size

Mitochondrial morphology using mitochondrial probes (HCQ001015; PKMito Orange; Biofount, Amsterdam, the Netherlands) was observed by multi-SIM. To quantify mitochondria size in SIM images, the area, perimeter and aspect ratio of individual mitochondria were measured using ImageJ (US National Institutes of Health, Bethesda, MD, USA). Morphometric analyses of mitochondrial were undertaken from at least three randomly selected views per group.

### 5.12. RNA interference (RNAi) experiments

Specific siRNAs targeting CCTα and CHKα were purchased from Keygen Biotech (Nanjing, China). siRNAs were transfected using Lipofectamine 3000 (Invitrogen) and OPTI-MEM (Gibco, Grand Island, NY, USA) according to manufacturer instructions.

### 5.13. AcCoA measurement

AcCoA in cells and tissues was measured using the Acetyl-CoA Assay Kit (ab87546; Abcam) according to manufacturer protocols. Briefly, cells were added to the assay buffer and homogenized, then deproteinized using tri-cellulose Acetate (TCA). Tissues were added to the assay buffer and TCA, and homogenized. After centrifugation (12,000 × *g*, 15 min, 4°C), the supernatant was neutralized with saturated KOH (1 M) until pH = 7-8 and centrifuged (120,000 × *g*, 5 min, 4°C). Standard curves for AcCoA were generated using AcCoA standards (0-1,000 pmol/mL). Fluorescence was measured using an excitation wavelength of 535 nm and emission wavelength of 589 nm. Background values were eliminated from the standards, and the concentrations of samples were calculated using a spectrophotometer (BioTek).

### 5.14. FFA measurement

FFAs in cells and tissues were measured using the Free Fatty Acid Assay Kit (ab65341; Abcam) according to manufacturer protocols. Briefly, cells and tissues were homogenized in 200 μL of chloroform/Triton X-100 (1% Triton X-100 in pure chloroform), followed by incubation on ice for 10-30 min. Centrifugation (12,000 × *g*, 10 min, 4°C) was followed by air-drying at 50°C in a fume hood to remove chloroform. \After vacuum-drying for 30 min to remove trace levels of chloroform, the organic (lower) phase was collected. Next, 200 μL of Fatty Acid Assay Buffer was used to dissolve dried lipids, followed by vortex-mixing extensively for 5 min. Standard curves for FFAs were generated using different concentrations. We measured the output immediately on a microplate reader at an absorbance of 570 nm. Background values were eliminated from the standards, and the concentrations of the samples were calculated using a spectrophotometer (BioTek).

### 5.15. PC measurement

PC in cells, serum, and tissues was measured using a kit (MAK049-1KT; Merck, Rahway, NJ, USA) according to manufacturer protocols. Briefly, cells and tissues were homogenized in 200 μL of lysis buffer, then centrifuged (12,000 × *g*, 10 min, 4°C). PC levels in serum samples were measured directly. Supernatants were extracted for subsequent analyses. Standard curves for PC were generated using different concentrations. We measured the output immediately on a microplate reader at an absorbance of 570 nm. Background values were eliminated from the standards, and the concentrations of samples were calculated using a spectrophotometer (BioTek).

### 5.16. Subcutaneous and intracranial xenotransplantation of GBM cells

Female Balb/c nude mice (20 g) were purchased from Beijing HFK Bioscience (Beijing, China). Mice were kept in a pathogen-free animal facility. For experiments using the intracranial tumor, BALB/c-nu/nu mice were inoculated subcutaneously with 1×10^6^ U251 MG cells. Palpable tumors (50 mm^3^) developed after 7 days. Then, mice were divided randomly into three groups of seven. The three groups received intraperitoneal injections of vehicle, CHA (20 mg/kg), or CHA (40 mg/kg) once a day for 14 days, respectively. The tumor diameter was measured with a caliper every 3 days. The tumor volume (mm^3^) was estimated using the formula of length × width^2^/2. After killing, tumors were dissected and immobilized in formalin for immunohistochemical analyses. Data are the mean ± SD of seven mice in each group. The orthotopic xenograft model of glioma was achieved using 5×10^5^ U87 MG cells and U87 MG stable cell lines (shACAT1#7, shACAT1#9; Vector). Briefly, cells were injected 2 mm to the right and 0.8 mm to the anterior bregma and 3.5 mm below the skull in nude mice. After 3 days, mice were divided randomly into three groups and injected with vehicle, CHA (20 mg/kg), or CHA (40 mg/kg) once a day for 14 days. The groups of U87 MG stable cell lines were observed for 14 days without any treatment. Mice were monitored daily and killed when neurological symptoms were observed. Their brains were dissected and immobilized in formalin for staining (hematoxylin and eosin).

### 5.17. MRI

After 11 days of treatment, a small-animal MRI scanner (Pharma Scan 70/16 US; Billerica, MA, USA) was used to acquire anatomical images of intracranial tumors. Choline measurement in *ACAT1^-/-^* mice also involved use of an MRI scanner. Prior to imaging, mice were anesthetized with inhaled isoflurane. The parameters used in the scans to optimize gray matter/white matter contrast were a T2_TurboRARE, with time of repetition/echo time = 5000/40, six averages, field of view of 20 × 20, and slice thickness of 0.5 mm. The scanning of all brains was completed in 14 sessions.

### 5.18. MS

We used an ultrahigh-performance liquid chromatography (UHPLC) system (1290 Infinity II) coupled to a quadrupole time-of-flight (Q-TOF) mass spectrometer (6550 iFunnel) equipped with a dual AJS electrospray ionization source (Agilent Technologies, Santa Clara, CA, USA). Phosphatidylserine (145849-32-7), CDP-Diacylglycerol (799812-77-4), and Phosphatidyl ethanolamine (384835-53-4) standards were purchased from Avanti Biosciences (San Diego, CA, USA). U87 MG cells (shACAT1 #1 and shACAT1 #2; Vector) were cultured simultaneously. Cells were collected when the cell density was 80%. They were washed thrice with phosphate-buffered saline, and pre-cooled 80% methanol solution was added to the culture dish. Cells were scraped off gently with a cell spatula and stored at -80°C for 12 h, then crushed by ultrasound. Cells were blown dry in nitrogen, and 150 µL of methanol (including the internal standard) was used for resolution. The supernatant was taken after centrifugation for MS. Bicinchoninic acid was used for protein quantification.

### 5.19. Statistical analyses

Results are the mean ± SD. Data were analyzed using the Student's *t*-test or one-way ANOVA. Multiple comparisons were made with Dunnett's test. Significance was defined as p < 0.05.

## Supplementary Material

Supplementary figures.

## Figures and Tables

**Table 1:** Primer sequences

Primer name	Forward primer (5′-3′)	Reverse primer (5′-3′)
*Nestin*	CTGCTACCCTTGAGACACCTG	GGGCTCTGATCTCTGCATCTAC
*SOX2*	CACATGAACGGCTGGAGCAA	GGAGTGGGAGGAAGAGGTAAC
*GFAP*	ACATCGAGATCGCCACCTAC	ACATCACATCCTTGTGCTCC
*S100β*	TGGCCCTCATCGACGTTTTC	ATGTTCAAAGAACTCGTGGCA
*Galc*	TATTTCCGAGGATACGAGTGGT	CCAGTCGAAACCTTTTCCCAG
*OLIG2*	CCAGAGCCCGATGACCTTTTT	CACTGCCTCCTAGCTTGTCC
*NeuN*	CCAAGCGGCTACACGTCTC	CGTCCCATTCAGCTTCTCCC
*β3-Tubulin*	GGCCAAGGGTCACTACACG	GCAGTCGCAGTTTTCACACTC
*ACAT1*	TACCAGAAGTAAAGCAGCATGG	TACCAGAAGTAAAGCAGCATGG
*GAPDH*	GTGGACCTGACCTGCCGTCT	GGAGGAGTGGGTGTCGCTGT

## Abbreviations

ACAT1: Acetyl coenzyme A acetyltransferase
AcCoA: Acetyl-CoA
ATRA: All-trans retinoic acid
APL: acute promyelocytic leukemia
CCT: Cytidylyltransferase
CDPCHO: Cytidine-5'-diphosphate choline
CHA: Chlorogenic acid
CHKα: Choline kinase alpha
DAG: Diacylglycerol
FAO: Fatty acid oxidation
FFA: Free fatty acid
GBM: Glioblastoma
PDH: Pyruvate dehydrogenase
PDK1: Pyruvate dehydrogenase kinase 1
PDP1: Pyruvate dehydrogenase phosphatase1
PS: Phosphatidylcholine
