# Supplementary material for: ACAT1 Induces the Differentiation of Glioblastoma Cells by Rewiring Choline Metabolism
Source: Int J Biol Sci. 2024 Oct 14;20(14):5576–93. doi: 10.7150/ijbs.96651 (PMC11528465; doi:10.7150/ijbs.96651)
Supplement: Supplementary file 1 — Supplementary figures. [file ijbsv20p5576s1.pdf]

Supplemental information

ACAT1 Induces Glioblastoma Cells Differentiation by Rewiring Choline Metabolism

Running title: ACAT1 Determines Glioblastoma Cells Differentiation.

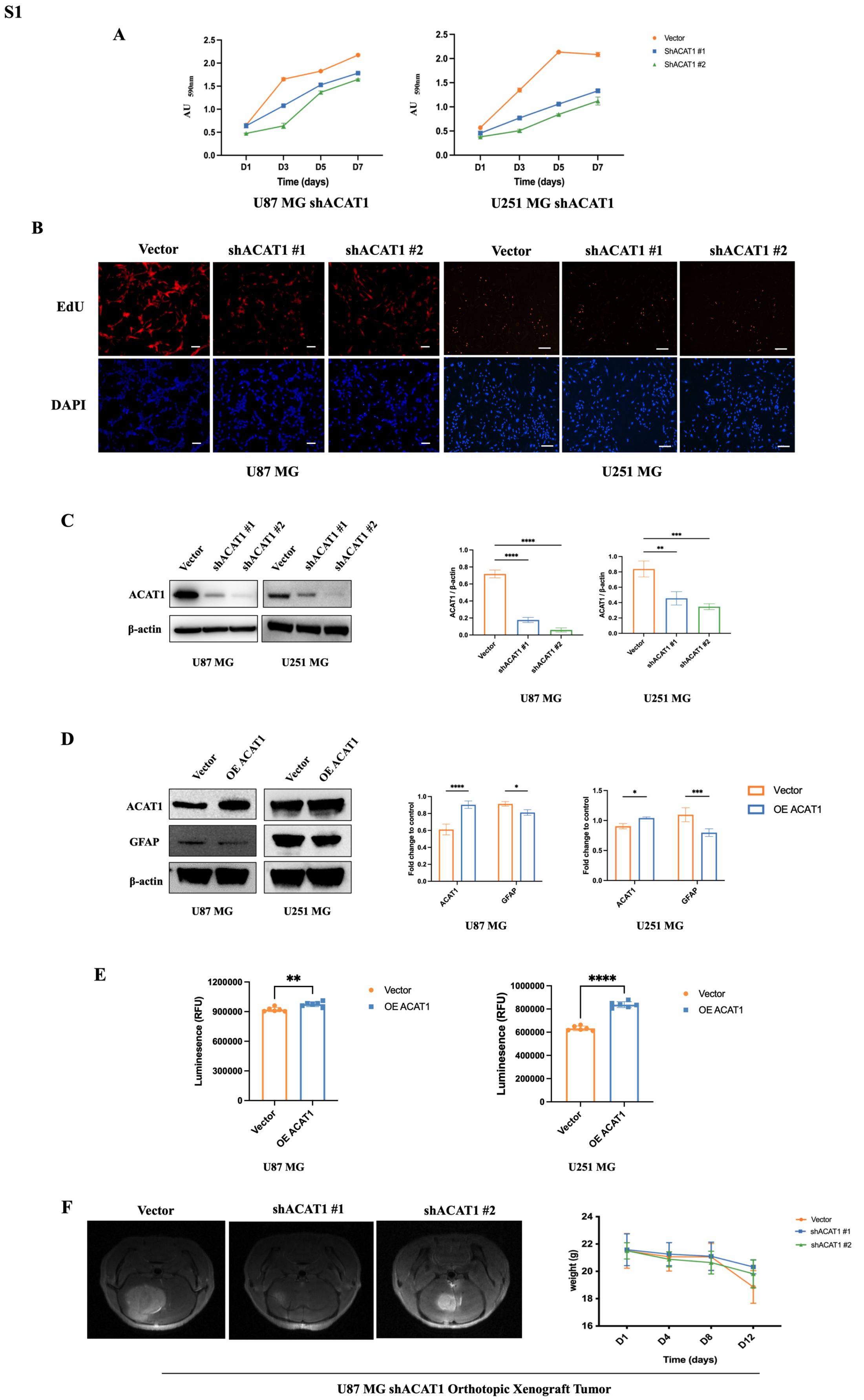
S1
A
AU 590nm
Time (days)
D1
D3
D5
D7
U87 MG shACAT1
U251 MG shACAT1
Vector
ShACAT1 #1
ShACAT1 #2
B
Vector
shACAT1 #1
shACAT1 #2
EdU
DAPI
U87 MG
U251 MG
C
ACAT1
β-actin
ACAT1 / β-actin
U87 MG
U251 MG
D
Vector
OE ACAT1
ACAT1
GFAP
β-actin
Fold change to control
U87 MG
U251 MG
E
Luminesence (RFU)
Vector
OE ACAT1
U87 MG
U251 MG
F
Vector
shACAT1 #1
shACAT1 #2
weight (g)
D1
D4
D8
D12
Time (days)
U87 MG shACAT1 Orthotopic Xenograft Tumor

Figure S1. ACAT1 KD in GBM cells inhibited the proliferation and migration of cells. (A) Cell-proliferation curve of ACAT1 KD in GBM cells. (B) The percentage of proliferating EdU-positive cells were measured by flow cytometry in U87 MG and U251 MG cells of shACAT1. The left scale bar was 100 µm, and the right one was 200 µm. (C–D) Western blotting was done to determine the change of ACAT1 in ACAT1 KD and ACAT1-overexpressed GBM cells. (E) Cell proliferation was detected by CTG. (F) Tumor volume using MRI. Changes in bodyweight of animals after inoculation with the U87 MG shACAT1 stable-transfer cell line.

S2

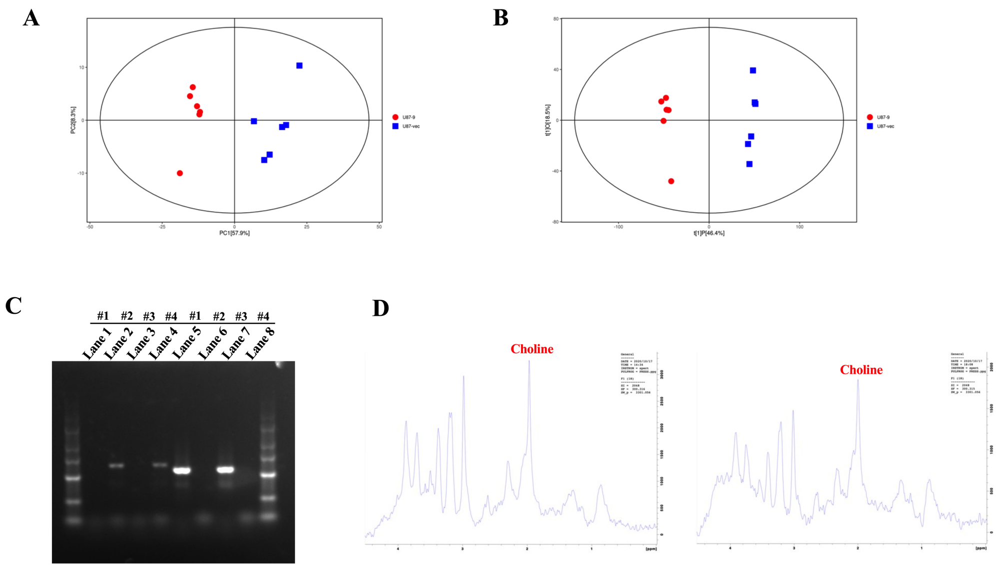

Figure S2. PCA and PLS_DA plots were used for the metabolomics analysis of ACAT1 KD GBM cells. (A–B) PCA analysis is on the left, and the right is the OPLS-DA score plot. (C) Genomic DNA extracted from mice tails, PCR followed by DNA gel electrophoresis, and gel imaging under UV. Lanes 1–4 denote F1R1 amplification (*ACAT1*$^{wild\ type}$). Lanes 5–8 show F1R2 amplification (*ACAT1*$^{-/-}$). That is, #1 and #3 were identified as *ACAT1*$^{-/-}$ mice, and #2 and #4 were identified as *ACAT1*$^{wild\ type}$ mice. (D) Choline identified in 1H NMR of *ACAT1*$^{-/-}$ mice.

S3

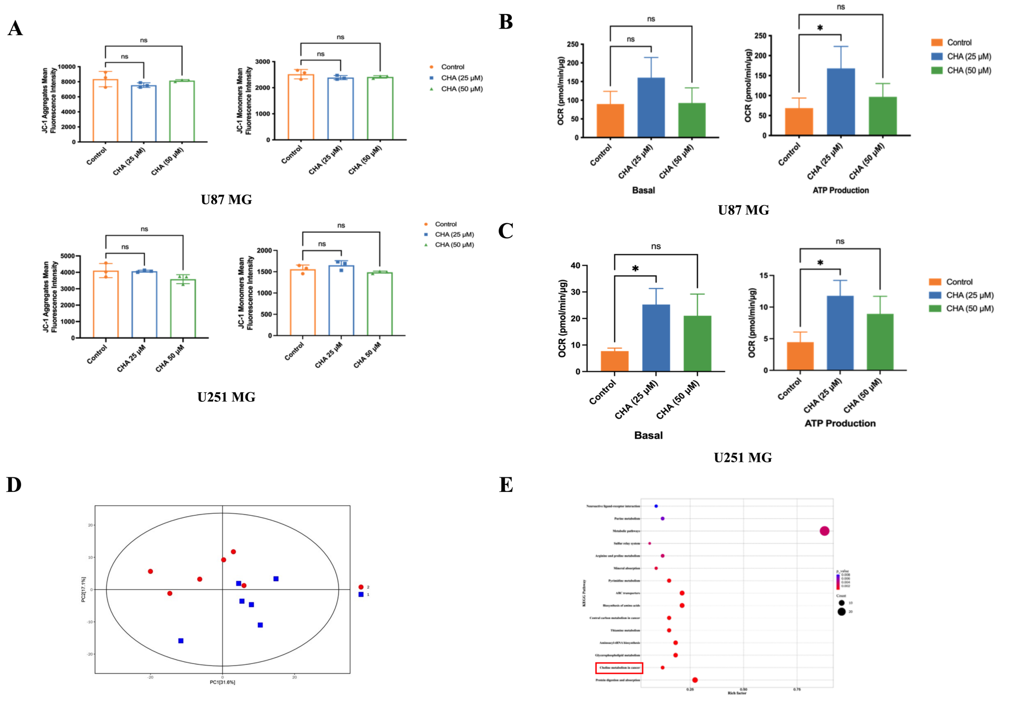

Figure S3. CHA activated the choline metabolic pathway and promoted the differentiation of GBM cells. (A) The mitochondrial membrane potential of GBM cells treated or not treated with CHA (25 μM or 50 μM, 24 h) was determined by JC-1 staining. (B–C) OCR was determined using GBM cells treated with CHA (25 μM or 50 μM) for 7 days. (D) PCA for the metabolomics analysis of U 87 MG cells treated with CHA (50 μM) for 7 days. (E) Analysis of signaling-pathway enrichment using the KEGG database indicated activation of the choline metabolic pathway in GBM cells after 1 week of CHA treatment.

S4

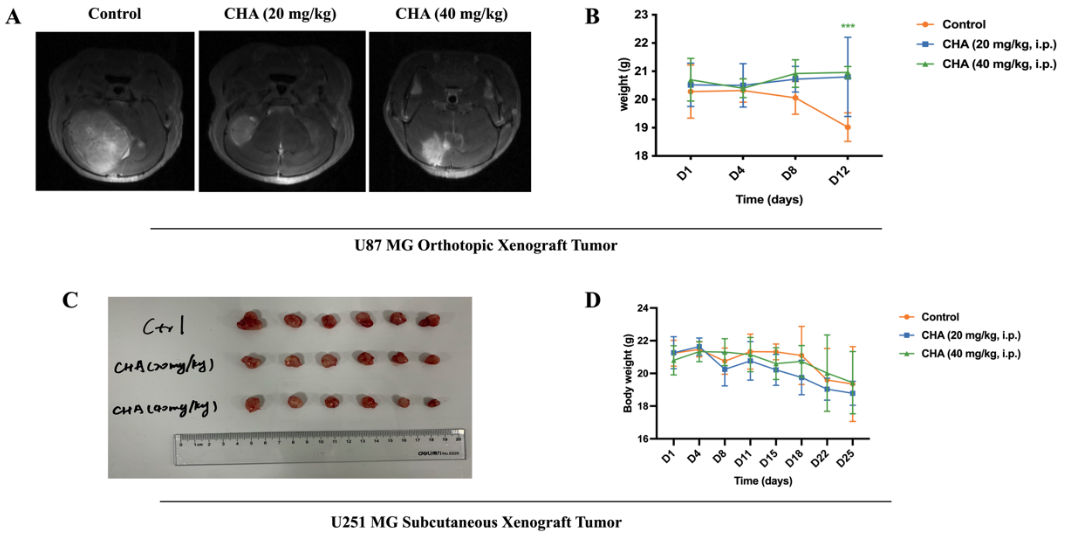

Figure S4. CHA inhibited the proliferation of GBM cells *in vivo*. (A–B) Tumor volume of U87 MG cells obtained from MRI scans. Bodyweight was recorded every 3 days. (C–D) Subcutaneous tumors of U251 MG cells were removed and photographed after animals had been killed. Bodyweight was measured every 3 days.
